# Applying the behaviour change technique (BCT) taxonomy v1: a study of coder training

**DOI:** 10.1007/s13142-014-0290-z

**Published:** 2014-11-19

**Authors:** Caroline E Wood, Michelle Richardson, Marie Johnston, Charles Abraham, Jill Francis, Wendy Hardeman, Susan Michie

**Affiliations:** 1 UCL Centre for Behaviour Change, Research Department of Clinical, Educational and Health Psychology, University College London, 1-19 Torrington Place, London, UK; 2 Health Services and Population Research, Kings College London, London, UK; 3 Institute of Applied Health Sciences, University of Aberdeen, Aberdeen, UK; 4 University of Exeter Medical School, University of Exeter, Exeter, UK; 5 School of Health Sciences, City University London, London, UK; 6 Primary Care Unit, Institute of Public Health, University of Cambridge, Cambridge, UK

**Keywords:** Behaviour change techniques, Taxonomy, Training methods

## Abstract

Behaviour Change Technique Taxonomy v1 (BCTTv1) has been used to detect active ingredients of interventions. The purpose of this study was to evaluate effectiveness of user training in improving reliable, valid and confident application of BCTTv1 to code BCTs in intervention descriptions. One hundred sixty-one trainees (109 in workshops and 52 in group tutorials) were trained to code frequent BCTs. The following measures were taken before and after training: (i) inter-coder agreement, (ii) trainee agreement with expert consensus, (iii) confidence ratings and (iv) coding competence. Coding was assessed for 12 BCTs (workshops) and for 17 BCTs (tutorials). Trainees completed a course evaluation. Methods improved agreement with expert consensus (*p* < .05) but not inter-coder agreement (*p* = .08, *p* = .57, respectively) and increased confidence for BCTs assessed (both *p* < .05). Methods were as effective as one another at improving coding competence (*p* = .55). Training was evaluated positively. The training improved agreement with expert consensus, confidence for BCTs assessed, coding competence but not inter-coder agreement. This varied according to BCT.

## INTRODUCTION

Effective interventions aimed at changing health behaviours of individuals, communities and populations are needed to improve health and reduce the prevalence of disease [1, 2]. Such interventions are often complex and comprise several potentially interacting active components [3]. This can make them challenging to accurately replicate in research, to synthesise across studies in evidence reviews and to translate into practice. Thus, to inform the development of more effective health behaviour change interventions and to enhance the understanding of their mechanisms of action, it is crucial that researchers report interventions with clarity and detail.

In the last decade, several guidance documents have been published aimed at improving methods of specifying and reporting interventions in published reports. For example, CONSORT (The Consolidated Standards of Reporting Trials [4, 5]) and TREND statements (Transparent Reporting of Evaluations with Nonrandomised Designs [6]) and the UK Medical Research Council’s (MRC) evaluation framework [7, 8]. CONSORT advises researchers to report the ‘precise details’ of the intervention as ‘actually administered’. In contrast to interventions in biomedicine, no standardised language exists for reporting the ‘active components’ delivered in behaviour change interventions [9]. For example, different labels are sometimes used to identify the same techniques, and different techniques may be identified by the same label (e.g. behavioural counselling). The precise ‘active ingredients’ of interventions are often therefore difficult to establish.

To address this gap and to provide a more rigorous methodology for characterising intervention content, researchers have begun to specify the active ingredients of interventions in terms of their component behaviour change techniques (BCTs). BCTs are defined as the observable, replicable components of behaviour change interventions. They are the smallest components compatible with retaining the proposed mechanisms of change and can be used individually or in combination with other BCTs [10–12]. Goal setting, self-monitoring of behaviour and action planning are all examples of BCTs.

The first cross-behaviour classification system to demonstrate inter-coder reliability in identifying 22 BCTs and 4 BCT packages in descriptions of interventions was published in 2008 [13]. Building on this and five other taxonomies [14–18], Michie and colleagues developed BCT taxonomy v1 (BCTTv1); the first cross-behaviour, hierarchically organised taxonomy. It was established by international expert consensus and comprises 93 clearly labelled, well-defined behaviour change techniques with demonstrated reliability in specifying 26 of the most frequently occurring BCTs [10, 12].

Identifying (coding) BCTs involves a deductive process of categorising qualitative information (e.g. descriptions of interventions) using an established coding framework and instructions. The process of coding BCTs is a highly skilled task requiring familiarity with the BCT labels and definitions and one which involves coders making a series of complex interpretative judgments [19, 20]. Achievement of good inter-coder reliability (i.e. the extent to which coders agree on the presence/absence of BCTs identified in intervention descriptions using the taxonomy as a coding framework) is therefore not only a function of the clarity of the taxonomy and its coding guidelines but also of the competences of its coders.

To maximise the reliability and confidence of using the taxonomy, coders should be trained to reliably recognise BCTs as defined by the taxonomy rather than relying on their own subjective judgements [21]. Inter-coder reliability has been demonstrated in using BCT taxonomies amongst coders with varying amounts of training and experience [13, 15, 16, 18, 22, 23]. Where reported, training in taxonomies has mainly involved manual-based coding instructions, provision of one-to-one feedback from taxonomy developers and prompting of coding practice. The intensity and the delivery of training has varied with some coders receiving intensive one-to-one feedback from developers and others training themselves by working through a coding manual. Systematic development and documentation of the training process and evaluation, involving the comparison of coding competence to apply BCT taxonomies both reliably and with accuracy before and after training, will establish whether systematic training can enhance coding competence.

One objective of BCT training is to teach users to recognise a BCT as it is defined in the taxonomy rather than relying on their own subjective judgements which might be triggered by the BCT label [21, 24]. It is additionally important to assess whether training enhances the ‘validity’ of coder judgements: the extent to which coders agree with BCTs agreed on as present or absent by expert BCT coders. Expert coding, assessed here as a consensus between expert BCT coders, is the closest we have to an objective standard of ‘validity’. An effective training programme, therefore, would be one that not only enhances inter-coder agreement between trainees but that also enhances agreement with expert consensus about BCTs identified.

The research literature suggests that collaborative or cooperative training strategies (i.e. working together in small groups towards a common goal) and active learning techniques, such as discussion, are more effective than traditional, lecture-style training for acquiring new knowledge, building skills and increasing motivation for improving new skills [25]. An effective training programme is built on four basic principles: (1) setting of training goals involving provision of information or concepts to be learned, (2) demonstration of knowledge and skills to be learned, (3) practice or rehearsal of skills learned and (4) provision of feedback to trainees during and after practice [26, 27]. Skills are more likely to be retained and improve future practice if trainees feel challenged, receive positive feedback and find the learning process interesting and enjoyable [27, 28]. Coder training incorporating these principles and BCTs has previously been evaluated in using the taxonomy to specify BCTs in written descriptions of behavioural support in smoking cessation [29]. This study found that training delivered in a short, 3-h workshop, delivered to a mix of research psychologists and non-psychologist practitioners significantly improved coding competence in terms of their agreement with expert consensus about which BCTs were present.

The popularity of the BCT approach (in particular the specification of interventions using BCT taxonomies) has prompted high demand for training in the reliable and valid application of BCTTv1. In response to this, two methods of training (workshops and tutorials) were developed based on previous BCT coder training conducted within the research teams of the study investigators and the established principles of learning and coding listed above. They were designed to train coders to accurately identify the most frequently occurring BCTs from BCTTv1, i.e. those which were found to occur most frequently in intervention descriptions. The decision was made to train and assess coders in the most frequently occurring BCTs as training 93 BCTs was not feasible within the proposed timeframe nor was it practical for trainees to learn at such a high level of intensity. Additionally, it was decided that frequently occurring BCTs would be more accessible to trainees as well as more useful for them to learn. Two methods of training were developed: workshops, which involved face-to-face group training for 1 day, and group-based distance tutorials, which were delivered via teleconference call to groups of two to four trainees in four, 1-h sessions held over 4–8 weeks. Tutorial training enabled training of coders internationally. This paper presents two sub-studies which report an initial evaluation of the effectiveness of these training methods and address the following research questions:

1. Does face-to-face training (1-day workshops) and distance training (group tutorials) improve the reliable specification of behaviour change interventions by BCT as assessed by increased:
  (i) Inter-coder agreement about BCTs identified
  (ii) Agreement with BCTs identified by expert consensus
  (iii) Confidence ratings for BCTs identified as present
2. Do trainees evaluate BCTTv1 training as a useful experience?
3. What proportion of trainees reaches an acceptable standard of competence following training?

## ONE-DAY WORKSHOPS

### Method

#### Design

Coding competence was assessed before and after each workshop by coding assessment tasks. To overcome potential practice effects, the assessments were administered in a counter-balanced design so that a random 50 % of trainees completed assessment task A at the beginning of the workshop and task B at the end, and the other half of trainees completed task B followed by task A. Trainee experience of training was assessed at the end of the training in a confidential evaluation questionnaire.

#### Participants

Participant details are presented in Table 1. Workshops were offered to those interested in investigating, reviewing, designing or delivering behavioural interventions; no previous knowledge or experience was required. They were advertised via scientific and professional organisations and the BCT Taxonomy Project website (http://www.ucl.ac.uk/health-psychology/bcttaxonomy/). Five workshops were conducted for the groups of between 9 to 29 trainees (*n* = 109).

**Table 1:** Demographic characteristics of workshop and group tutorial trainees

		Workshops	Tutorials
		*N* = 109	*N* = 52
Age (mean (SD))		32.31 (9.27)	37.04 (7.82)
Gender	Female	103	35
	Male	6	17
Profession^a^	Practitioner	12	7
	Student	57	3
	Academic	40	40
Highest qualification	BA/BSc	23	–
	MA/MSc	46	10
	PhD	35	33
	Clinical	4	9
Nationality	UK	72	38
	European (non-UK)	22	8
	America	8	4
	Asia	5	–
	Australia	1	1
	South Africa	1	–
Previous experience of taxonomy use *N* (%)	Coding Describing BCIs	26 (24) 35 (32)	28 (54) 36 (69)
Expertise associated with BCIs (mean (SD))^b^	Designing	2.38 (1.10)	3.60 (1.00)
	Delivering	2.46 (1.22)	3.19 (1.04)
	Reporting	2.45 (1.16)	3.51 (0.87)
	Reviewing	2.46 (1.13)	3.45 (1.08)
	Using behaviour change theories	3.30 (0.90)	3.70 (0.77)

^a^Data was unavailable for two tutorial trainees

^b^Response scale ranging from 1 (none) to 5 (a great deal)

#### Materials

##### Training

Coding manuals of previous taxonomies [12, 13] were used to inform the development of the workshop training programme. Trainees were taught 24 of the frequently identified BCTs from BCTTv1. Training involved trainees watching three short PowerPoint presentations and participating in a series of interactive coding exercises as a group, individually and in pairs. Content was structured around a series of learning objectives (e.g. ‘to learn the need for precise BCT labels and definitions’, ‘to avoid wrongly inferring the presence of a BCT’) and was designed in terms of BCTs that aimed to positively influence coding behaviour and changing skilled behaviour (e.g. graded tasks, behavioural practice/rehearsal, instruction on how to perform the behaviour, feedback on behaviour; for a full list of BCTs used, see Table 2). Workshop tasks were delivered according to a number of different formats: via a ‘ready, steady, point!’ task for which trainees were shown a short excerpt on the presentation screen and asked, when prompted, to point to the left if BCT X was present, to the right if BCT Y was present or to the ceiling if there were unsure (see Table 2). Tasks increased in difficulty as the workshop progressed from simple tasks and coding short excerpts through to more difficult tasks involving the coding of longer, more complex excerpts. Each 1-day workshop was delivered by two experienced BCT coders (BCTT project team members).

**Table 2:** Summary of 1-day workshops content and learning objectives

Content		Brief description	Learning objectives	BCTs used
Presentation	Background to Behaviour Change Techniques	Outlines the goals for the day, defines and conceptualises the term ‘BCT’ and communicates need for agreed standard list of BCTs in behavioural medicine	• To understand aims, objectives and learning outcomes • To learn need for precise labels and definitions	• Credible source • Social reward • Pros and cons • Comparative imagining of future outcomes
Assessment	Using the taxonomy	Trainees work individually to identify presence/absence of 12 BCTs in an intervention description	• To assess pretraining use of BCTs	• Instruction on how to perform the behaviour • Behavioural practice/rehearsal
Task	Ready, steady, point!	Trainees shown a short excerpt on screen and, when prompted, asked to point left BCT1, right for BCT2 or to the ceiling if unsure	• To learn BCTs 1–2 labels and definitions • To learn appropriate levels of inference and discrimination	• Social comparison • Salience of consequences • Graded task • Behaviour practice/rehearsal • Feedback on behaviour
Morning break				
Presentation	Identifying behaviours and BCTs	Defines behaviour and the distinction between behaviour and behaviour outcome	• To learn what a behaviour is and the difference between a behaviour and a behaviour outcome	• Behaviour practice/rehearsal • Feedback on behaviour • Generalisation of target behaviour • Social comparison • Feedback on behaviour • Social reward
Task	Identifying behaviours and BCTs	Trainees work in pairs to highlight exact words showing behaviour and BCT in two short excerpts	• To learn how to identify BCT 3–6 in written text • To consider descriptions in detail	• Behaviour practice/rehearsal • Feedback on behaviour
Task	Identifying BCTs in reports	BCTs 7–12 cards are placed around the room. Trainees shown five excerpts taken from real interventions; for reports one and two, trainees asked when prompted, to point at the correct BCT card. For reports three to five, trainees asked to identify presence of BCTs 7–12	• To consolidate previous learning of BCTs 1–12 • To learn how to reliably identify BCTs in real and increasingly complex reports	• Social reward • Instruction on how to perform the behaviour • Behaviour practice/rehearsal
Task	Providing examples of BCTs	Trainees work in groups of four to generate their own examples of BCTs 7–12 and then feedback to the rest of the group	• To learn BCTs through detailed consideration of examples	
Lunch break				
Task	Role play	Expert tutors act out two role plays with six of BCTs 13–24 in each. Trainees asked to identify presence of BCTs	• To learn BCTs 13–24 labels and definitions and recognise them as delivered in practice	• Behaviour practice/rehearsal
Task	Coding published descriptions	Trainees work in pairs to identify presence of BCTs 1–24 in two longer excerpts	• To consolidate previous learning of BCTs 1–24 • To consolidate previous learning of coding for presence of BCTs and making inferential judgements in real reports	• Graded tasks • Feedback on behaviour • Demonstration of the behaviour • Social reward • Feedback on outcome
Afternoon break				
Presentation	Moving from a list to a taxonomy	Introduces the idea of a hierarchical structure and outlines how it was developed	• To become familiar with hierarchical structure of the taxonomy and the 24 BCTs within their clusters	• Problem solving
Assessment	Using the taxonomy	Trainees work individually to identify presence/absence of 12 BCTs in an intervention description	• To assess post-training use of BCTs	• Feedback on behaviour (if requested)

#### Measures

##### Trainee’s previous experience

Trainees were asked if they had previously: (1) designed or reported behaviour change interventions that specifically identified BCTs, (2) been involved in writing manuals or protocols of interventions, and (3) undertaken a narrative or systematic review of behaviour change literature. They also rated their expertise (i.e. knowledge, skills and familiarity) in the areas of designing, writing, reporting and systematic reviewing of behaviour change interventions using response options from 1 (‘no experience’) to 5 (‘a great deal of experience’).

##### Evaluating training effectiveness in increasing coding competence

Coding competence for 12 BCTs was assessed before and after training. Trainees were asked to identify the presence/absence of BCTs in descriptions of two behaviour change interventions targeting increasing physical activity and increasing safe needle cleaning behaviour, respectively. They were also asked to rate how confident they were of their identification using +/++, whereby ‘+’ represented ‘BCT present in all probability but evidence not clear’ and ‘++’, ‘BCT present beyond all reasonable doubt; clear evidence available’. The intervention descriptions used were written (by CA and MR) to highlight the learning principles taught and to ensure the inclusion of the frequent BCTs covered by the training.

To assess trainees’ agreement with expert consensus, six highly experienced BCT coders who had been involved in developing BCTTv1 (study team members: MJ, SM, JF, WH, CA and MR) worked in pairs (which were randomly allocated) and independently coded the descriptions using BCTTv1. Expert consensus was developed by the discussion of any discrepancies within each of the pairs. SM and the study researcher (MR) reviewed remaining discrepancies where a resolution was not immediately obvious. The list of BCTs agreed on as a result of this process was then circulated to the whole study team to agree the final BCT codings. We used this consensus as a criterion against which trainee coders’ codings were compared and validity was assessed. Consensus was reached about the presence of 12 BCTs in the descriptions: self-monitoring of behaviour, feedback on behaviour, behavioural practice/rehearsal, nonspecific reward, goal setting (outcome), material reward (behaviour), credible source, problem solving, demonstration of the behaviour, information about health consequences, goal setting (behaviour) and social support (unspecified).

Training effectiveness was evaluated by changes in inter-coder agreement, in trainee agreement with expert consensus, in the proportion of high (i.e. ++) confidence ratings for the 12 BCTs assessed and in the proportion of trainee coders reaching an acceptable standard of competence. Agreement (both inter-coder and with expert consensus) was assessed using prevalence and bias-adjusted kappa (PABAK) [30, p. 425] (see Analysis) and acceptable standard of competence was defined in terms of trainee agreement with expert consensus (for rationale, see “Analysis”).

##### Evaluating trainee experience of training

To evaluate and to inform improvement and optimisation of future training methods, trainees rated the usefulness of the presentations and individual and group tasks (i.e. ‘ready, steady, point!’ tasks, identifying BCTs in published reports and identifying BCTs in role plays; see Table 2 for more details) in helping them to build skill and knowledge, using response options from 1 ‘not useful’ to 5 ‘useful’. All trainees were asked to respond to four open-ended questions: (1) what part(s) of the training did you find the most useful? (2) what part(s) or aspect(s) of the training, if any, did you find least useful? (3) would you like future training? If so, do you have a specific proposal? and (4) please provide any other feedback about using BCT taxonomies.

#### Procedure

Prior to attending the workshop, all trainees were sent two articles as preparatory reading [10, 11]. At the beginning of the workshop, they were asked to provide demographic information (i.e. age, gender, nationality, professional background and highest qualification). All trainees completed an assessment of their coding competence before and after training and completed a training evaluation questionnaire. They received a BCTTv1 training certification at the end of the workshop and individual feedback on their coding competence assessments via email.

## DISTANCE GROUP-BASED TUTORIALS

### Method

#### Design

Training was conducted over four, 1-h sessions. Training was held over an average period of 6 weeks with a minimum of 1 week in between each tutorial session. Each tutorial group was led by an experienced BCT tutor. Experienced BCT tutors (*N* = 10) included five members of the BCTTv1 study team (MJ, SM, WH, JF and MR) and five experts in behaviour change. The five experts had previously been involved as part of their own research and practice on at least one occasion, in designing and reporting behaviour change interventions which identified BCTs, in writing manuals of interventions, had undertaken systematic or narrative reviews of behaviour change interventions or had published behaviour change studies. For 9 out of the 10 groups, tutorial sessions took place via teleconference call. Trainees’ coding competence was assessed before and after training by assessment tasks and trainees’ evaluation of training (in terms of usefulness) was assessed after training in an evaluation questionnaire.

#### Participants

Participant details are presented in Table 1. Tutorial training was advertised via the same networks as 1-day workshops. BCTT project team members were also asked to identify colleagues from their own teams/networks. Those interested in taking part were asked to complete a self-evaluation form. Trainees (*n* = 52) were invited to join the training if they indicated they had some previous experience in investigating, designing and/or delivering behaviour change interventions and were available over the training period. As a greater level of commitment was required from tutorial trainees (i.e. commitment to attend four tutorial sessions and completion of work in their own time followed by a study task), trainees were recruited from those who expressed an interest in taking part in the training and subsequent study task. They were offered an honorarium on completion of the study task. Trainees were contacted and recruited via email, with the offer of an honorarium of £560 on completion of a coding task for research purposes (estimated to take 2 days) following the group tutorial training programme [31].

#### Materials

##### Training

Training was structured around the same BCTs, learning principles and objectives as workshops (see Table 3) with the content adapted to cover a wider range of 44 BCTs from BCTTv1. Content was delivered according to a training manual. Coding manuals of previous taxonomies [13, 26] were used to inform the development of the manual. Training was piloted in a face-to-face format with one group of trainees and these data were included in the analyses. The other nine tutorial sessions were conducted via teleconference call.

**Table 3:** Summary of learning principles and learning objectives for group-based tutorials

Tutorial session	Learning principle introduced in the session	Learning objectives
1	Only code BCTs that are directly applied to the target behaviour(s) and population(s)	• To understand and accurately apply General coding instructions (8 preliminary steps) and Learning principle 1 • To reliably identify the presence/absence of BCTs 1–10
2	Do not infer the presence of a BCT. The description must correspond to the definition of the BCT given in the taxonomy. If you are unsure, do not code the BCT as present	• To consolidate understanding and accurate application of General coding instructions (8 preliminary steps) and Learning principle 1 • To understand and accurately apply Learning principle 2 • To reliably identify the presence/absence of BCTs 1–20 in longer, more complex pieces of text
3	Take care distinguishing between BCTs that only differ in terms of their behaviour change type (i.e. behaviour vs. outcome)	• To consolidate understanding and accurate application of general coding instructions (eight preliminary steps) and learning principle 1 and 2 • To understand and accurately apply Learning principle 3 • To understand the need for clear BCT labels and definitions • To reliably identify the presence/absence of BCTs 1–32
4	Code technical terms and packages of BCTs that map onto BCTs in the taxonomy	• To consolidate understanding and accurate application of General coding instructions (eight preliminary steps) and learning principle 1, 2 and 3 • To understand and accurately apply Learning principle 4 • To reliably identify the presence/absence of BCTs 1–44 in longer more complex pieces of text

Each tutorial group had four trainees who were paired into two sets of ‘buddies’. The purpose of the buddy system was to provide trainees with both practical and emotional forms of *social support* during their training and to foster independent and shared *problem solving*. Prior to each session, coders independently completed and submitted a preparatory coding task (comprising one, two or three exercises dependent on tutorial session) to their tutor before discussing with their buddy. Each buddy pairing was asked to discuss their task in advance identifying key questions and issues they wanted to raise during the tutorial session. Trainees were encouraged to log the outcome of their buddy conversation in their ‘learning log’ to maximise the usefulness of the tutorial session and to help identify areas for discussion and reflection. Tutors assessed their groups’ completed coding tasks and compared trainee coding to consensus coding (i.e. BCTs consensually agreed on as present by experienced BCT coders). Tutors led a discussion of the discrepancies between trainee and expert coding during the tutorial session.

#### Measures

##### Trainee’s previous experience

Previous experience was assessed as for workshops.

##### Evaluating training effectiveness in increasing competence

Trainees were asked to identify the presence/absence of BCTs in intervention descriptions before and after training. Two descriptions were used: one for the before training assessment task and one for the after training assessment task. The target behaviour for both interventions was increasing healthy eating and physical activity. Descriptions were written (by CA and MR) to highlight the learning principles and to ensure inclusion of the frequent BCTs taught in training. Expert consensus was reached about the presence of 17 BCTs from BCTTv1 in each of the two descriptions: feedback on behaviour, credible source, information about health consequences, social support (practical), information about social and environmental consequences, social support (unspecified), monitoring of outcome(s) of behaviour without feedback, nonspecific reward, demonstration of the behaviour, Adding objects to the environment, goal setting (behaviour), problem solving, self-monitoring of outcome of the behaviour, goal setting (outcome), behavioural practice/rehearsal, self-monitoring of behaviour, instruction on how to perform the behaviour and material reward (behaviour).

Trainees were provided with a training taxonomy (a shortened version of BCTTv1 comprising 44 BCTs) and coded the presence/absence of BCTs in the descriptions, rating their confidence in each BCT identification using the same +/++ ratings as for workshops.

Training effectiveness was evaluated by changes in inter-coder agreement, agreement with expert consensus and in the proportion of high (i.e. ++) confidence ratings for the 17 BCTs assessed. As an additional measure of effectiveness, tutorial trainees completed measures of perceived confidence and reported intentions to use a taxonomy to code reports and describe behaviour change interventions, using response options ranging from 1 (‘strongly disagree’) to 5 (‘strongly agree’), before and after training.

##### Evaluating trainee experience of training

Using the same response options as for 1-day workshops, trainees rated perceived usefulness of the reading materials provided prior to the first tutorial session, the materials provided for sessions 1 to 4, the content and the structure of the sessions and the preparatory coding tasks completed prior to each session, the buddy system and the learning log. They completed the same open-ended evaluation items as for workshops.

#### Procedure

Prior to their first tutorial session, trainees were sent the same two preparatory reading articles and completed the same measures (i.e. demographic information, experience in the use of BCTs and BCT taxonomies) as for workshops. Tutorial trainees were also asked to watch a short, introductory PowerPoint presentation on the advantages and challenges associated with the use of the BCT approach in specifying the content of behaviour change interventions. All trainees completed the coding competence assessment task (see “Measures”) before training. After their final tutorial session, trainees completed the after training coding competence assessment task and a training evaluation questionnaire. Trainees received individual feedback on their coding competence assessments and more generic feedback on some of the common coding discrepancies their group and that other groups had across the four tutorial sessions. Trainees received their feedback and a BCTTv1 training certificate via email.

### Analysis

The following analyses were performed on data collected from workshops *and* tutorials.

*Inter*-*coder agreement about BCTs identified* was assessed by using PABAK [30]. PABAK was used rather than Cohen’s kappa [32] or percentage agreement as it adjusts for potential chance agreement between coders and high prevalence of negative agreement (i.e. when both coders agree the BCT is absent). For the purposes of analysis, trainees were randomly allocated into coding pairs across workshop/tutorial groups using a random number generator. Coder pairings were kept the same for both before and after training analyses. Where both trainees identified the BCT as present or absent, agreement was recorded and where one trainee identified the BCT but the other did not identify the BCT, disagreement was recorded. PABAK was calculated for each pair and means reported across pairs and for each of the BCTs assessed. The percentage of coding pairs achieving good reliability (i.e. a PABAK score of .60 and above) before and after training was also calculated. To assess improvement in inter-coder agreement for each of the BCTs assessed, the frequency of agreements and disagreements between coding pairs was calculated for each BCT and entered into a Fisher’s exact test (to allow for cell counts of less than five) [33].

*Trainee agreement with expert consensus* was assessed by calculating the number of BCTs identified by each trainee that were also identified by expert consensus. The number of agreements and disagreements between trainees and experienced BCT coders were recorded and were used to calculate PABAK. Means were reported across trainee-expert consensus pairings and for each of the BCTs assessed. Where a trainee identified the BCT identified as present by expert consensus, agreement was recorded and where the trainee did not identify the BCT, or identified a BCT not included in the consensus, disagreement was recorded. To assess improvement in trainee agreement with expert consensus for each of the BCTs assessed, the frequency of agreements and disagreements between coding pairs was calculated for each BCT and entered into a Fisher’s exact test (to allow for cell counts of less than five) [33].

*Confidence for BCTs identified* was assessed by calculating the frequency and percentage of high confidence ratings (i.e. ‘++’: BCT present beyond all reasonable doubt; clear evidence available) for BCTs identified as present. High confidence ratings were included in the analysis so that we could easily distinguish BCTs identified with certainty. The percentage of high confidence ratings for each BCT was also reported to identify which of the BCTs assessed was identified with confidence by the greatest percentage of trainees. The threshold was set at 90 %. This decision to use this value was arbitrary and served only to highlight the greatest percentage of trainees identifying with confidence.

*Change following training* was assessed using paired samples *t* tests to assess change in the following: (i) agreement between trainees about BCTs identified (inter-coder PABAK), (ii) the number of BCTs identified by trainees also agreed on as present by expert consensus, (iii) trainee agreement with expert consensus (trainee-consensus PABAK) across trainee-consensus pairings and across the BCTs assessed, (iv) high confidence ratings (i.e. ‘++’) for BCTs identified as present, (v) perceived confidence and (vi) reported intention to use BCT taxonomies in the future. Paired *t* tests were used as exploratory data analysis testing for normality indicated that the distributions for all variables did not deviate significantly from that of a normal distribution (all *p*’s < .05).

#### Trainee’s previous experience

Frequencies and percentages were calculated to describe the number and proportion of trainees with previous experience of BCT taxonomy use. Means and standard deviations were calculated to describe trainee’s expertise associated with behaviour change interventions (separate means were calculated for each of designing, delivering, reporting, reviewing experience and for use of behaviour change theories; an overall mean was calculated across these categories).

#### Evaluation of training

Means and standard deviations were used to summarise trainee ratings of the content of the training and ratings of the materials used. A content analysis of the written feedback was conducted by two researchers (CW and KS) to identify training components that participants found useful or not so useful; CW read the feedback generated by trainees and conducted the first round of analysis; KS checked allocation to content categories. Discrepancies were resolved through discussion between the two researchers.

#### The proportion of trainees reaching an acceptable standard of competence following training

We conceptualised an acceptable standard of competence as being the extent to which individual trainees agreed with expert consensus. Landis and Koch [34] suggested that kappa values of .60–.79 indicate ‘substantial’ reliability with those above .80, indicating ‘outstanding’ reliability. We used this as a guide and considered that trainees achieving a PABAK score of .60 and above to have reached an acceptable standard of competence. A chi-squared test was used to explore the proportion of workshop and tutorial trainees reaching the competence criterion, before and after training. We assessed the effectiveness of workshop and tutorial training in increasing competence using a 2 (before vs. after training) × 2 (workshop vs. distance tutorial) analysis of variance.

## RESULTS

### Evaluating effectiveness of training in increasing coding competence

One-day workshops (see Tables 1 and 4)

**Table 4:** One-day workshops: before and after training agreement between coders, coder agreement with expert consensus and confidence in BCT Identification

BCT number and label	Before training				After training			
(BCTs ordered according to mean PABAK inter-coder agreement between trainees, after training)	*N* trainees identifying BCT (max = 109)	Mean PABAK agreement between trainees	Mean PABAK trainee agreement with expert consensus	% trainee identifications with high confidence ratings (i.e. ++)	*N* trainees identifying BCT (max = 109)	Mean PABAK agreement between trainees	Mean PABAK trainee agreement with expert consensus	% trainee identifications with high confidence ratings (i.e. ++)
2.3. Self-monitoring of behaviour	49	.49	−.10	22	101	.96	.85	88
2.2. Feedback on behaviour	56	.49	.03	57	93	.93	.71	92
8.1. Behavioural practice/rehearsal	81	.42	.49	80	98	.70	.80	90
10.3. Nonspecific reward	74	.48	.36	86	75	.56	.38	88
1.3. Goal setting (outcome)	60	.64	.22	89	84	.56	.54	87
10.2. Material reward (behaviour)	104	.56	.91	85	105	.48	.93	88
9.1. Credible source	68	.20	.25	57	75	.44	.38	87
1.2. Problem solving	82	.45	.50	89	71	.33	.30	89
6.1. Demonstration of the behaviour	76	.27	.39	89	100	.30	.83	93
5.1. Information about health consequences	86	.67	.58	91	77	.19	.41	87
1.1. Goal setting (behaviour)	86	.93	.58	89	62	.19	.14	78
3.1. Social support (unspecified)	89	.78	.63	86	47	−.11	−.13	94

BCT labels and numbers correspond to BCTTv1 (Michie et al. [12])

#### Trainee’s previous experience

Participant details are presented in Table 1.

#### Inter-coder agreement between trainees

A trend indicated that average inter-coder agreement increased (*t*(54) = 1.77, *p* = .08) (before training: mean PABAK = .39, SD = .34; after training: mean PABAK = .50, SD = .26). Inter-coder agreement increased for 6 of the 12 BCTs assessed: self-monitoring of behaviour, feedback on behaviour, behavioural practice/rehearsal, nonspecific reward, credible source and demonstration of the behaviour (average increase in PABAK for these six BCTs = .26; SD = .18; range = .03–.47). However, change across the 12 BCTs was nonsignificant, *t*(11) = .90, *p* = .39. The number of agreements for the BCT material reward (behaviour) was high before training, leaving little scope for improvement. Reliability was maintained after training. There was a significant increase in the number of agreements between trainee coders for demonstration of the behaviour (*p* < .05) but significant decreases for the BCTs social support (unspecified) and credible source (both *p*’s < .05).

#### Agreement with expert consensus

Trainee agreement with expert consensus increased (*t*(108) = 3.26, *p* < .05) (before training: mean PABAK = .39, SD = .29; after training: mean PABAK = .50, SD = .28). Across the BCTs assessed, agreement increased for eight of the 12 BCTs: self-monitoring of behaviour, behavioural practice/rehearsal, nonspecific reward, feedback on behaviour, goal setting (outcome), material reward (behaviour), credible source and demonstration of the behaviour (average increase in PABAK for these eight BCTs = .36; SD = .33; range = .02–.95). However, change across the 12 BCTs was nonsignificant (*t*(11) = .56, *p* = .59). The number of trainee agreements with expert consensus for the BCT material reward (behaviour) was high before training, leaving little scope for improvement. Agreement with expert consensus was maintained after training. Significant increases were seen in the number of agreements between trainee coders and expert consensus for goal setting (outcome), demonstration of the behaviour, feedback on behaviour, behaviour practice/rehearsal and self-monitoring of behaviour (all *p*’s < .05). Significant decreases were seen for the BCTs goal setting (behaviour) and social support (unspecified) (both *p*’s > .05).

#### Confidence for BCTs identified

The number of high confidence ratings (i.e. ‘++’) that trainees assigned increased (*t*(108) = 4.89, *p* < .05) (before training: mean number of BCTs = 8.38, SD = 1.91; after training: mean number of BCTs = 9.56, SD = 1.93). Across the BCTs assessed, the number of high confidence ratings also increased (*t*(11) = 2.89, *p* < .05). The number of high confidence ratings increased for 6 of the 12 BCTs: feedback on behaviour, behavioural practice/rehearsal, self-monitoring of behaviour, credible source, material reward, demonstration of the behaviour. One of the BCTs, information about health consequences was rated with high confidence before training, by over 90 % of trainees. Four of the BCTs, demonstration of the behaviour, material reward (behaviour, behavioural practice/rehearsal and self-monitoring of behaviour, were rated with high confidence after training, by over 90 % of trainees.

Distance group-based tutorials (see Tables 1 and 5)

**Table 5:** Distance group tutorials: before and after training inter-coder agreement, trainee agreement with expert consensus and confidence ratings

BCT number and label (ordered according to mean PABAK inter-coder agreement between trainees, post-training)	Before training				After training			
	*N* trainees identifying BCT (max = 52)	Mean PABAK inter-coder agreement between trainees	Mean PABAK trainee agreement with expert consensus	% Trainee identifications with high confidence ratings (i.e. ++)	*N* trainees identifying BCT (max = 52)	Mean PABAK inter-coder agreement between trainees	Mean PABAK trainee agreement with expert consensus	% Trainee identifications with high confidence ratings (i.e. ++)
10.2. Material reward (behaviour)	47	.62	.81	85	49	.77	.88	82
4.1. Instruction on how to perform behaviour	19	.00	−.27	63	44	.69	.69	82
2.3. Self-monitoring of behaviour	47	.62	.81	91	48	.69	.85	92
8.1. Behavioural practice/rehearsal	16	.08	−.38	63	47	.62	.81	66
2.4. Self-monitoring of outcome(s) of the behaviour	45	.46	.73	86	45	.46	.73	91
1.3. Goal setting (outcome)	42	.38	.62	88	45	.46	.73	91
1.2. Problem solving	49	.77	.88	78	42	.38	.62	71
1.1. Goal setting (behaviour)	49	.77	.88	76	40	.38	.54	78
12.5. Adding objects to the environment	6	.54	−.77	33	36	.38	.38	72
6.1. Demonstration of the behaviour	27	−.31	.04	78	40	.38	.54	83
10.3. Nonspecific reward	11	.46	−.58	27	10	.38	−.62	50
2.5. Monitoring of outcome(s) of behaviour without feedback	37	.31	.42	51	41	.31	.58	71
3.1. Social support (unspecified)	10	.54	−.62	50	43	.31	.65	72
5.3. Information about social and environmental consequences	4	.85	−.85	25	30	.23	.15	60
5.1. Information about health consequences	50	.85	.92	84	41	.15	.58	83
9.1. Credible source	28	−.38	.08	43	39	.15	.50	90
2.2. Feedback on behaviour	27	−.31	.04	74	19	.00	−.27	58

BCT labels and numbers correspond to BCTTv1 (Michie et al. [12])

#### Trainee’s previous experience

Participant details are presented in Table 1.

#### Inter-coder agreement between trainees

There was no change in overall inter-coder agreement from before to after training (*t*(25) = .57, *p* = .57). Across BCTs assessed, inter-coder agreement increased for 8 of the 17 BCTs: feedback on behaviour, goal setting (outcome), credible source, demonstration of the behaviour, behavioural practice/rehearsal, self-monitoring of behaviour, instruction on how to perform behaviour, material reward (behaviour) (average increase in PABAK for these eight BCTs = .38; SD = .26; range = .08 to .69) but change across the 17 BCTs was nonsignificant (*t*(16) = .28, *p* = .78). As for workshops, the number of agreements between trainee coders for the BCT material reward was high before training, leaving little room for improvement. The number of agreements between trainee coders increased for the BCTs information about health consequences and instruction on how to perform the behaviour (all *p*’s < .05). A significant decrease in the number of agreements was seen for the BCT information about social and environmental consequences (*p* < .05).

#### Agreement with expert consensus

Agreement with expert consensus increased (*t*(51) = 6.60, *p* < .05) (before training: mean PABAK = .57, SD = .11; after training: mean PABAK = .72, SD = .14). Across the BCTs assessed, trainee agreement with expert consensus also increased (*t*(16) = 2.35, *p* < .05). Agreement increased for 11 of the 17 BCTs: credible source, information about social and environmental consequences, social support (unspecified), monitoring of outcome(s) of behaviour without feedback, demonstration of the behaviour, adding objects to the environment, goal setting (outcome), behavioural practice/rehearsal, self-monitoring of behaviour, instruction on how to perform the behaviour, material reward (behaviour) (average increase in PABAK for these 11 BCTs = .62; SD = .50; range = .04–1.27). As for workshops, the number of trainee agreements with expert consensus for the BCT material reward (behaviour) was high before training, leaving little scope for improvement. The number of agreements was also high for the BCTs problem solving and self-monitoring of behaviour. Significant increases were seen in the number of agreements between trainee coders and expert consensus for information about social and environmental consequences, instruction on how to perform the behaviour, behaviour practice/rehearsal, demonstration of the behaviour and adding objects to the environment (all *p*’s < .05). Significant decreases were seen for the BCTs goal setting (outcome) and credible source (both *p*’s > .05).

#### Confidence for BCTs identified

The number of high confidence ratings (i.e. ‘++’) that trainees assigned did not change (*t*(51) = −.57, *p* = .57). The number of high confidence ratings also increased across 13 of the 17 BCTs assessed (*t*(16) = −3.40, *p* < .05). Confidence increased for 13 of the 17 BCTs: credible source, information about social and environmental consequences, social support (unspecified), monitoring of outcome(s) of behaviour without feedback, nonspecific reward, demonstration of the behaviour, adding objects to the environment, goal setting (behaviour), self-monitoring of outcome(s) of the behaviour, goal setting (outcome), behavioural practice/rehearsal, self-monitoring of behaviour and instruction on how to perform the behaviour. One of the BCTs self-monitoring of behaviour was rated with high confidence before training, by over 90 % of trainees. Four BCTs were rated with high confidence after training, by over 90 % of trainees: self-monitoring of outcome(s) of behaviour, self-monitoring of behaviour, goal setting (outcome) and credible source.

There was a significant increase in perceived confidence in using the taxonomy (before training: *M* = 3.42, SD = 1.00; after training: *M* = 4.08, SD = .56) (*t*(51) = −5.27, *p* < .001). Reported intention to use BCT taxonomies in the future remained high from before (*M* = 4.23, SD = .74) to after training (*M* = 4.16, SD = 1.02) (*t*(51) = .44, *p* = .66).

#### Proportion of trainees reaching an acceptable standard of coding competence

Of the 109 workshop trainees, 25 achieved a PABAK score (in terms of agreement with expert consensus) of .60 or above and therefore met the criteria representing an acceptable standard of coding competence before training and 50 met the criterion after training (*χ*2 (*df* = 1, *n* = 218) = 12.70, *p* < .05). Of the 52 tutorial trainees, 18 met the criterion before training and 41, after training (*χ*2 (*df* = 1, *n* = 104) = 20.72, *p* < .05). The proportion of trainees reaching the standard increased across workshop and tutorial training: 46 % of workshop trainees and 78 % of tutorial trainees achieved a PABAK score of .60 or above (workshops: mean PABAK = .74, SD = .09; tutorials: mean PABAK = .77, SD = .10). The change in coding competence was significant for both workshops and tutorials and that training methods were as effective as one another at increasing competence (*F*(1,318) = .35, *p* = .55). Of the 12 BCTs assessed in workshops, two met the competence criterion of .60 before training and five met the criterion after training (*χ*2 (*df* = 1, *n* = 24) = 1.82, *p* = .81). Of the 17 BCTs assessed in tutorials, seven met the competence criterion of .60 before training and eight met the criterion after training (*χ*2 (*df* = 1, *n* = 34) = .12, *p* = .73).

#### Evaluating trainees’ experience

Training was evaluated positively by trainees with all components receiving uniformly high ratings (on the scale of 1 to 5) in terms of usefulness (workshops: *M* = 4.62; SD = .68; range = 3–5; tutorials: *M* = 4.30, SD = .67; range 4 to 5). Trainees reported that the combination of practical tasks and the opportunity for structured discussion during the tutorial sessions was particularly useful. Many reported that the tutorial sessions offered an opportunity to learn the ‘consensus answers and the rationale behind coding’. They felt the sessions provided them with the chance to learn why specific BCTs had been coded in specific contexts and then ‘discuss any reasons for discrepancies with other members of the group’. The majority commented that having access to a wide range and number of excerpts taken from published reports provided a useful opportunity to practice their newly learned skills. Skills training activities such as using the taxonomy to code short excerpts and longer descriptions from published intervention reports were rated as useful opportunities to apply their skills learned over the course of the day. A few trainees reported that the ‘Ready, steady, point!’ exercises were a useful method to help increase ability to identify BCTs at speed. Whilst the majority agreed that the ‘Learning Log’ and ‘Buddy system’ components were both useful in principle, feedback suggested that trainees wanted more guidance on how to use them. For example, some found them difficult to put into practice due to time constraints. This was particularly an issue for trainees from different time zones.

## DISCUSSION

Training in using BCTTv1 in the form of 1-day workshops or group tutorials improved average trainee agreement with expert consensus and increased the proportion of trainees reaching an acceptable standard of coding competence, to 46 and 78 %, respectively. Not including BCTs with high agreement before training, the number of agreements between trainees increased for 9 % of BCTs in workshops and 12 % in tutorials and increased the number of trainee agreements with expert consensus for 40 % of BCTs in workshops and 33 % in tutorials. Training improved trainees’ confidence in coding BCTs. However, it did not improve agreement between coders about the presence and absence of BCTs in descriptions of behaviour change interventions. The opportunity to apply knowledge and new skill in a number of coding tasks followed by group discussion was evaluated as being a useful approach by trainees.

These data provide insight for the following reasons. First, we identified BCTs for which training was effective. This is where inter-coder agreement and agreement with expert consensus was poor before training but good after training, for example, behavioural practice/rehearsal. Second, we identified BCTs that can be reliably and accurately identified with limited training. The BCT material reward (behaviour) achieved good reliability across both methods, before and after training. Amongst tutorial trainees, the BCTs self-monitoring and self-monitoring of outcome(s) of behaviour also achieved good reliability before and after training. Third, we identified BCTs that consistently achieved poor reliability. That is, BCTs that achieved poor reliability before and/or after training, e.g. goal setting (outcome), social support (unspecified) and information about social and environmental consequences.

In order to decrease the number of BCTs falling into this final category may mean that further refinement of labels and examples is needed or that more intensive training is needed before trained coders are able to identify them with high reliability and validity. The learning curve may be steeper for some BCTs than for others. The plan is for an international consortium to consider the experiences of users and published data from BCTTv1 and release BCTTv2 in a number of years when there is sufficient evidence to support a new version. Drawing on the feedback from trainees, one approach could be to further clarify the distinction between similar BCTs in the same grouping, e.g. goal setting behaviour and goal setting outcome, social support practical and social support unspecified to help coders distinguish between different types of BCTs, i.e. ‘behaviour’ versus ‘outcome’ BCTs. We acknowledge that learning rates amongst trainees may differ and also that a certain amount of ‘unlearning’ may be required before trainees can achieve good reliability and validity. For example, reliability got worse for some BCTs and it was possible for inter-rater reliability to be high and remain high even when criterion validity was low. It may be that trainees need to change their interpretation of a specific BCT label as it is understood within their own discipline or experience to how the BCT is defined in BCTTv1. This may explain why some BCTs achieved poor reliability and validity before and/or after training, especially amongst tutorial trainees who started with a greater level of experience and expertise related to the use of BCT taxonomies. However, it may also suggest that these BCT definitions need further clarification. As BCTTv1 develops, it is likely that the number of BCTs that can be effectively trained will increase.

At a more general level, the data suggest that training was beneficial for different types of trainee and across different modes of delivery. Differences between the two training methods in the percentage of trained coders reaching an acceptable standard of coding competence may partly be due to differences in competence *before* training. One-day workshops required a lower level of commitment from trainees compared to the level required from tutorial trainees. Workshops may have therefore attracted trainees with lower personal involvement and consequently perhaps lower competence in BCT coding. Tutorial training required trainees to make a commitment of at least 6 weeks and would naturally attract trainees with a vested interest. The fact that tutorial training allows for consolidation of learning between sessions and discussion and meaningful engagement with other trainees may also account for the higher proportion of competent coders after training. To take training in use of BCTTv1 forward, an online training programme has been developed using the tutorial session model (see www.bct-taxonomy.com).

An effective training programme should not only increase ability but also trainee confidence in applying new knowledge and skill. Training increased confidence in identifying BCTs assessed for both methods of training, and tutorial trainees also reported increased overall confidence in using the taxonomy. One could infer that the learning environment provided by tutorials (i.e. a support network built over the period of multiple and regular sessions, increased time for reflection and practice) encouraged tutorial trainee confidence to increase more readily. The current study found an increase in confidence yet modest levels of inter-coder agreement and trainee agreement with expert consensus, after training. These data highlight that a highly confident coder is not necessarily a competent coder. We have carried out further work to explore how confidence and competence relate to one another [31]. Forty coders trained in use of BCTTv1 via distance group tutorial training completed a coding exercise whereby they identified BCTs in 40 intervention descriptions published in protocols. They completed the exercise again, 1 month later. We assessed inter-coder reliability, validity of their coding (i.e. coder agreement with BCT identifications consensually agreed on by the taxonomy developers), test retest reliability and coding confidence. Our analyses showed that inter-coder reliability and validity of BCT identification tended to be negatively correlated with coder confidence. This suggests that confidence is perhaps not such a useful indicator of accuracy of BCT identification. It is important to acknowledge that confidence in applying BCTTv1 requires learning the complexities and challenges it faces; an understanding which is likely to improve with practice and perhaps one which may not have been fully achievable in an intensive 1-day workshop. Future research should also examine how confidence changes over time and with further training experience.

Whilst empirical comparison of the two training methods was not the focus of this paper, some reflections are possible about the feasibility and acceptability of each approach. Workshops may be considered the more cost- and time-effective option given that a relatively large group of coders can be effectively yet intensively trained over the course of a day. However, small group tutorial training, delivered over a longer period of time provides a learning environment including a support network, time for practice and time for reflection between sessions.

It is important to remember that the assessment of training effectiveness was based on trainee coding of just one intervention description before and after training. The training materials used in 1-day workshops and tutorials were written to exemplify particular BCTs, to highlight the learning principles and to ensure inclusion of the frequent BCTs targeted for training. In general however, the quality of published intervention descriptions is poor with different BCTs described using different terminology and referred to using different terminology [35, 36]. As the use of BCT methodology increases, BCT content is likely to become much clearer, leading to increased reliability of identifying BCTs [29]. It is also important to acknowledge that our trainees were predominantly female and from the UK. We had hoped to reach a wide range of trainees across nationality and gender by advertising workshops and tutorials on BCTT project website. As the use of BCTT methodology increases, it will be possible to recruit a wider range of trainees to evaluate training effectiveness. Finally, it should be acknowledged that coder pairings (both inter-coder pairings and trainee-expert consensus pairings) were the same for analyses of data before and after training. In the ideal assessment of inter-coder reliability, coders would be compared with all other coders and a multi-rater kappa statistic would be used to analyse the resultant data. This type of analysis will be possible in the future using a multi-rater equivalent of the PABAK statistic. So that the analyses could be compared with previous work (e.g. [12]), it was decided that randomly allocating trainees into pairings and calculating PABAK would be the best approach to take in the current work.

To maintain good levels of accuracy and reliability, knowledge and skills should be maintained [37]. We are currently evaluating the long-term impact of BCT training [38] but would recommend that coders already trained in use of BCTTv1 regularly review training materials (to maintain and further develop their knowledge and skills) before using the taxonomy and check their reliability before beginning data extraction. To provide this opportunity, and in order to train new coders using BCTTv1, we have developed an open-access online BCTTv1 training course which can be accessed at www.bct-taxonomy.com. The online training programme is based on the tutorial training model, e.g. inclusion of practice coding tasks, feedback on completed coding tasks and assessments, the possibility for structured discussion led by an expert BCT tutor, access to a support network to foster continued learning and implementation of the taxonomy and access to a wide range of additional resources. Coders will be trained on a greater number of BCTs than those taught and evaluated in the current paper.
